# Plantar heel pain: an update of its aetiology and diagnosis

**DOI:** 10.1186/1757-1146-6-S1-O18

**Published:** 2013-05-31

**Authors:** Karl B Landorf, Andrew M McMillan, Hylton B Menz

**Affiliations:** 1 Department of Podiatry, La Trobe University, Melbourne, Australia; 2 Lower Extremity and Gait Studies Program, La Trobe University, Melbourne, Australia

## 

Plantar heel pain/plantar fasciitis is one of the most common musculoskeletal complaints of the foot. This presentation reviews some of the more important recent findings to update practitioners on the aetiology and diagnosis of plantar heel pain.

A critical, narrative review of key findings from recent research that our research group and other investigators have conducted relating to plantar heel pain.

Two main issues of interest have recently been investigated, including the role of heel spurs and diagnostic imaging. Firstly, recent research indicates that heel spurs – once thought to be an incidental, painless finding – may have a greater role in causing symptoms than previously thought. Secondly, medical imaging has an increasingly important role in the diagnosis of plantar heel pain, and has furthered our understanding of its aetiology. For example, recent power Doppler research that we have conducted revealed a vascular component to plantar fasciitis. These insights question what we know about plantar heel pain, and may have implications for how we manage the condition.

There has been much recent advancement in what we know about plantar heel pain, including the role of plantar heel spurs and the findings from diagnostic imaging. While these advancements have helped in our understanding of this common condition, there is still more research needed to unravel exactly what it is.

